# A Transdisciplinary Approach to Address Climate Change Adaptation for Human Health and Well-Being in Africa

**DOI:** 10.3390/ijerph18084258

**Published:** 2021-04-17

**Authors:** Caradee Yael Wright, Candice Eleanor Moore, Matthew Chersich, Rebecca Hester, Patricia Nayna Schwerdtle, Guy Kakumbi Mbayo, Charles Ndika Akong, Colin D. Butler

**Affiliations:** 1 Environment and Health Research Unit, South African Medical Research Council, 1 Soutpansberg Road, 0001 Pretoria, South Africa; 2 Department of Geography, Geoinformatics and Meteorology, University of Pretoria, 0001 Pretoria, South Africa; 3 School of Social Sciences, University of KwaZulu-Natal, 4041 Durban, South Africa; moorec@ukzn.ac.za; 4 Wits Reproductive Health and HIV Institute, University of the Witwatersrand, Klein Street, 2001 Johannesburg, South Africa; MChersich@wrhi.ac.za; 5 Virginia Tech, Department of Science, Technology, and Society, Blacksburg, VA 24060, USA; rjhester@vt.edu; 6 Nursing and Midwifery, Monash University, 3800 Clayton, Australia; patricia.schwerdtle@monash.edu; 7 Heidelberg Institute of Global Health, Heidelberg University, Im Neuenheimer Feld 672, 69120 Heidelberg, Germany; 8 World Health Organization, African Regional Office, P.O. Box 06, Brazzaville, Congo; mbayog@who.int (G.K.M.); cakong@who.int (C.N.A.); 9 National Centre for Epidemiology and Population Health, Australian National University, 2600 Canberra, Australia; bodhiaus@gmail.com

**Keywords:** climate change policy, disaster risk, early warning systems, environmental health, health governance, healthcare, sustainable development, transnationality

## Abstract

The health sector response to dealing with the impacts of climate change on human health, whether mitigative or adaptive, is influenced by multiple factors and necessitates creative approaches drawing on resources across multiple sectors. This short communication presents the context in which adaptation to protect human health has been addressed to date and argues for a holistic, transdisciplinary, multisectoral and systems approach going forward. Such a novel health-climate approach requires broad thinking regarding geographies, ecologies and socio-economic policies, and demands that one prioritises services for vulnerable populations at higher risk. Actions to engage more sectors and systems in comprehensive health-climate governance are identified. Much like the World Health Organization’s ‘Health in All Policies’ approach, one should think health governance and climate change together in a transnational framework as a matter not only of health promotion and disease prevention, but of population security. In an African context, there is a need for continued cross-border efforts, through partnerships, blending climate change adaptation and disaster risk reduction, and long-term international financing, to contribute towards meeting sustainable development imperatives.

## 1. Introduction

Health and climate change are increasingly being addressed together within policy frameworks. The overarching *2008 Libreville Declaration on Health and Environment* is the African political framework for approaches to address environment and health and includes climate change adaptation [1]. Under the auspices of this Declaration, the World Health Organization (WHO) supports Vulnerability Assessments, Situation and Needs Assessments and Health Adaptation Plans (HAPs). The WHO also supports African countries to submit National Adaptation Plans (NAPs) to the United Nations Framework Convention on Climate Change. NAPs comprise essential public health interventions from baseline risk and capacity assessment, to surveillance, partnership strengthening and research promotion. The WHO provides support to African countries to integrate health into NAPs and Nationally Determined Contributions (country-level plans to reduce national emissions and adapt to climate change impacts) [2].

Partnership is key in the WHO’s approach to climate change and health adaptation by supporting member states cojointly with the UN Environment and engaging other partners through the *Health and Environment Strategic Alliance* [3]. In 2017, the WHO released a *Regional Strategy for Environmental Determinants of Health (2017*–*2021)* to guide countries to reduce the burden of disease through safe, sustainable and health-enhancing human environments [4]. Climate change is one of the strategy’s principal focus areas. Some of the ways in which the health sector has engaged in climate change adaptation have included training of health personnel to deal with changing patterns of infectious and also noninfectious diseases, increasing geographical accessibility to health services and policy development, for example, mainstreaming climate change in health policies and strategies [5]. The current SARS-CoV-2 pandemic saw the Southern African Development Community unite to tackle COVID-19 through planning and preparedness and joint cooperation together with the WHO and the African Centres for Disease Control [6].

In November 2018, participants of the *Third Interministerial Conference on Health and Environment* prepared a strategic action plan to scale up health and environment interventions between 2019 and 2029 in Africa [7]. Fast development and implementation of HAPs were emphasised. Some HAPs included the need for improved surveillance systems to address emerging health risks, building the evidence base on climate change and health, and increasing capacity in the health system. Others did not include actionable details on the role of the health sector for climate change adaptation [5,7]. Yet, while partial roles for health sectors in African countries to act and engage with climate change adaptation activities have been identified, we propose a wider approach than previously considered, incorporating meaningful consultation, and grounded on available literature and case studies [8,9].

## 2. Shifting our Thinking about Health and Climate Change Adaptation in Africa

In Africa, climate change continues to increase warming over land and regions, alter rainfall patterns and water availability, and affect ecosystems [10]. These changes, among several others, multiply existing health vulnerabilities, including insufficient access to safe water and improved sanitation, food insecurity and limited access to healthcare and education. Further, socio-political and biological governance structures are generally bounded by nation-state concerns and borders. Yet the health issues related to climate change, for example, variable temperatures and extreme weather, vector-borne illnesses, and population displacement, are issues that do not respect human-made geographic and political borders. As climate change increases the burden of climate-relevant health outcomes [11] “*reforming global governance for health and improving the capacity of low- and middle-income countries (LMICs) to negotiate and safeguard their interests*” [12], on their own, are necessary but not sufficient as effective solutions. We argue that a more effective response necessitates a holistic, transdisciplinary [13,14], multisectoral and systems approach that “thinks” health governance and climate change together in a transborder framework. This broad health-climate approach would require novel thinking regarding geographies, ecologies, socio-economies and policies, and it would centre on the most vulnerable populations. It would also need to tackle some challenging issues, such as sovereignty, procurement and international financing mechanisms.

Africa is currently one of the most vulnerable regions to climate change, and its populations are among the most mobile in the world [15]. Rather than continuing to work from siloed institutional, sectoral or national frameworks, therefore, governance responses should account for the increased cross-border movement of people by addressing health-climate issues within a regional frame that acknowledges and works with the social networks and established historical relationships that transcend borders [16]. In Africa, border demarcations reflect the whims of colonial powers, rather than cultural bonds or climate zones, as elsewhere in the world. Governance efforts at the health-climate nexus need to respond to transborder biological and ecological phenomena brought about by climate change, such as vector-borne illness or livestock diseases, in a more regional manner. In many instances, nation-state-based systems are not equipped to deal with changing patterns of mobility in the context of climate change, such as internal displacement and trapped populations, and their health consequences [17]. Research in Africa demonstrates that political tensions have been exacerbated by scarcity of food and water [18]. Civil conflict tied to climate variability [15], and subsequent mass migration, is expected to increase [19]. An example of climate change amplifying the risk of conflict in fragile states is the 2003 conflict in Darfur, South Sudan. Climate and environmental change multiplied pre-existing tensions between farming villagers and pastoralists as rainfall and vegetation declined and seasonal migratory patterns changed. The government used these tensions to bolster its support among the ethnic groups it favoured. This incited the conflict that involved a high-level of violence directed at civilians [20,21].

Although increased violent conflict or state failure are most often linked to climate change, the health risks posed by climate-related population movements are a major source of human suffering, disability and loss of life [22]. For example, African refugees and migrants face various health risks along the migration route, including lack of access to health services, water, food and shelter along the way, as well as dangerous transportation and terrain. Between January and June 2016, the International Organization for Migration (IOM) had recorded 471 deaths and disappearances in Africa, many due to exposure, hunger or dehydration in the Sahara Desert. Cross-border migrants, such as Zimbabweans living in Botswana and South Africa, also experience challenges once they arrive at their destination, including lack of access to health services driven by a fear of deportation, the long distances to health facilities, the negative attitudes of health workers, the cost of travel, a lack of time, as well as a poor understanding of disease [23]. International migrants and refugees likewise experience high rates of morbidity and mortality as exemplified by the high death rates of Sub-Saharan African migrants in France, especially around Paris, compared to the broader French population, during the COVID-19 pandemic [24].

Under current projections of sustained emissions and unequal development, we can anticipate an increase in multidimensional problems, including climate change-associated conflict, that may render populations more vulnerable and lead to increased displacement [25] disproportionately impacting LMICs. Health governance institutions and policies within and beyond these countries need to be prepared to respond to heightened levels of displacement and migration. One way to achieve this is to develop more regional initiatives that will allow for service delivery, access to medicines, health communication and health financing across borders. The United States Agency for International Development (USAID) Climate Links Adaptation Thought Leadership and Assessment [26] provides a range of examples. For example, the USAID Famine Early Warning Systems Network is a malaria early warning system in Senegal and Kenya to inform early identification and communication of potential malaria outbreaks [27]. The WHO through their regional offices, together with other key stakeholders, could facilitate additional cross-border initiatives by convening meetings, providing training and capacity building, and facilitating financing mechanisms that transcend nation-state-based agendas. *The African Transport Policy Program* is an example of the latter [28].

## 3. Interconnectedness to Address the Health-Climate Change Emergency

**Figure 1:**
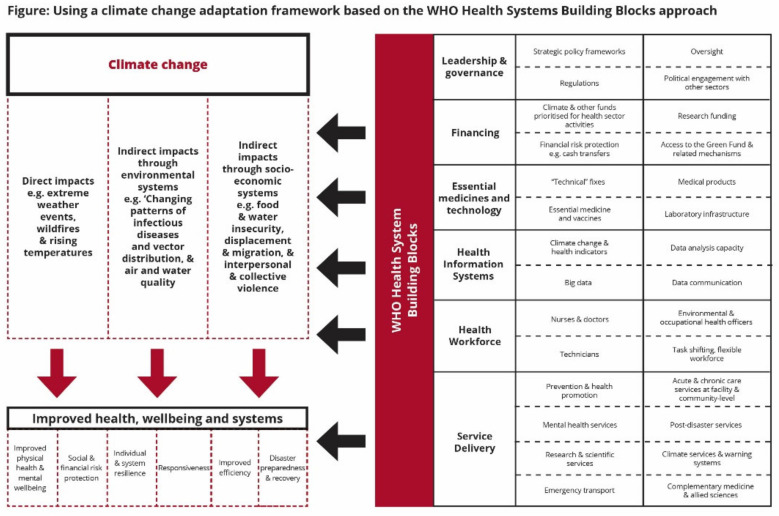
Unpacking a climate change adaptation framework based on the WHO Health Systems Building Blocks.

Direct and indirect impacts of climate change on health and well-being are mediated by social, economic, cultural, behavioral and geopolitical determinants of health and well-being. A range of exposure pathways including heat stress, air quality and vector ecology, lead to various health impacts [29]. An example of a health impact mediated through the water quality and quantity exposure pathway in Ethiopia is diarrhoeal disease. Diarrhoeal deaths in children under 15 years old that are attributable to climate change are projected to significantly increase under a high emissions scenario. Accordingly, diarrhoeal disease has been identified as a priority climate-sensitive disease in Ethiopia [30]. Many virulent infections are also highly climate-sensitive. Humidity, temperature and precipitation exert a strong influence on vector life cycles, and the infectious agents they carry, and influence the transmission of vector borne diseases. Both malaria and dengue have been identified as priority climate sensitive diseases in Ghana [31]. Climate change-related interventions are required to strengthen the health system (Figure 1) [32,33]. Efforts underway include, for example, transborder laboratory networks for testing and diagnosis of infectious disease [34], and the *African Collaborative for Health Financing Solutions* [35], a project that supports African countries to advance toward universal health coverage, for example, with targeted training for policymakers.

The interconnectedness of health sector components illustrates the complexities that need to be unpacked to meet holistic health-climate adaptation [9]. Much like a ‘Health in All Policies’ approach [36], one should think health governance and climate change together in a transnational framework as a matter not only of health promotion and disease prevention, but of human and population security. While the WHO building blocks do not necessarily lend themselves to a transdisciplinary approach, they reflect the foundations upon which most healthcare systems are built, and the widely used analytical framework. Appling this framework could promote alignment between health systems and emerging climate change response.

Climate change is considered to be the greatest global health threat of the century [37] not only because of its direct impact on morbidity and mortality as a result of heat waves and infectious disease, for example, but principally through its impact on indirect pathways such as food insecurity and conflict [38]. Influenced by the same reasoning, many nations have framed climate change as a national security threat, preparing for major social and economic upheaval. Yet, research shows that the public is more motivated to mitigate climate change when it is framed as a health issue, with messages delivered by health professionals [32], rather than as a security issue [37]. Building on these and similar important findings, our approach incorporates such a comprehensive health-climate lens.

Continued efforts, through partnerships, blending climate change adaptation and disaster risk reduction, long-term international financing including Global Environmental Funds and the Green Climate Fund, as well as international and domestic private sector contributions through green operation footprints and funding injections, are needed [7]. Humanitarian and development organizations play an important role in health service provision in fragile and failed states, and are adapting operations through mitigation, adaptation and advocacy strategies to be better equipped to anticipate, prevent, prepare for and manage climate-related health risks [9,38,39].

Many climate change-related impacts on health stem from complex interacting issues and adaptative responses and thus need to account for factors beyond the health sector. Impacts of climate change on the agricultural sector, for example, affect food safety and security, as well as many people’s income, employment and mental health. As has been documented in Sub-Saharan Africa, “the intensity and frequency of extreme climatic events can cause mental health disorders including post-traumatic stress disorder, depressive disorders, anxiety, and other serious conditions” [40,41]. Therefore, to adapt against health impacts of climate change, variability or disasters, it is critical to take a broad approach that includes other sectors. For example, the close dependency of people in Africa on rainfed agriculture, and the surrounding human-animal ecosystems, forms a context where integrated approaches such as One Health or the Planetary Health framework explicitly consider the ecological, economic and social foundations of health, including indigenous and local knowledge [42,43].

While many actions already undertaken by the health sector in Africa provide protection against climate-sensitive conditions, including optimising coverage of childhood and other vaccines [44] and reducing vector-borne infections through insecticide-treated bed nets [45], concerted transborder, transdisciplinary and multisectoral holistic action is required to strengthen institutional capacity for risk monitoring and early warning systems, emergency preparedness and response, vulnerability reduction measures, shock-responsive and long-term social protection, and planning and implementing resilience building measures [9]. Health status depends on access to healthcare, but also on economic circumstances, employment rates, infrastructure such as roads, and educational systems. Deficiencies in many of these factors are especially common in Africa and addressing these, in addition to health system’s deficits, are key to reducing the vulnerability to climate change on the continent. Moreover, adaptation strategies are necessarily context-specific, tailored to the specific health conditions related to climate change in an area, community norms and resource constraints. The adaptation response by the health sector alongside all other sectors in Africa, as across the globe, needs to centre on providing holistic services for individuals and groups at higher risk of adverse health outcomes due to climate change.

## 4. A Geopolitical, Transdisciplinary Approach

The WHO *Regional Strategy for Environmental Determinants of Health* looks at developing “win-win” solutions (similar to the ‘no/low regrets’ approach by the Intergovernmental Panel on Climate Change) through sustainable development choices that also mitigate climate change. In addition to improving air pollution and advocating for a greener healthcare sector, development of strategies and building capacities that enhance sustainable development while also improving health, governance and social capacity across borders, are needed. Already underway, the WHO’s *Special Programme for Research and Training in Tropical Diseases* is promoting the research agenda on climate change and health.

We might also consider developing strengthened health governance solutions that respond to emerging geopolitical realities magnified or shaped by climate change, such as heightened population mobility. The COVID-19 pandemic is having enormous health impacts, and also devastating economic and social effects that have subsequent negative health consequences. Analogously, climate change, if left unchecked, threatens significant direct and indirect adverse effects, damaging health, eroding resilience and undermining security and livelihoods. It is vital that the health sector finds an inclusive approach that emphasizes cross-border collaboration and respects human rights while also mitigating the morbidity and mortality of the forcibly displaced. One possible way may be to research morbidity pathways and design ecosystem-based interventions to reduce climate-exacerbated morbidity in Africa [45].

While there will be many challenges to achieving the approach that we advocate, the comprehensive policy infrastructure for the approach described here already exists in nascent form in many of Africa’s five geopolitical regions and under the auspices of the African Union. For example, 47 African Health Ministers endorsed in 2019 the *Framework on the Implementation of the Global Vector Control Response* [46]. This strategy is designed to strengthen collaboration between governments across sectors, engage and mobilize communities and to enhance surveillance and scale up and integrate approaches to managing vector-borne disease. However, when the complexity of some of the continent’s emergencies is considered, such steps, while significant, may not be enough. Malnutrition, for example, increases the risk of contracting and dying from diseases such as malaria, and is one of the indirect impacts of climate change on health outcomes in the region [47]. Furthermore, the financial burden of addressing crises such as these are sometimes beyond the reach of single governments, and their efforts, and those of nongovernment organizations assisting them, may be made more effective by pooling resources and data across nation states, although we recognize that significant political barriers exist to such resource sharing. There are also lingering challenges with integrating or coordinating donor funding. The *Global Fund to Fight AIDS, Tuberculosis and Malaria* provides a useful model for international financing and partnerships in LMICs.

## 5. Conclusions

Due to the interrelatedness of the social, political, economic and biological issues generated by climate change, and because of the gaps in current approaches, in this short communication we have advocated a creative approach to health governance that focuses both on mitigating the synergistic effects that climate change will have on human-animal-environmental health and on achieving synergistic policy effects through building collaborative health governance infrastructures. A transnational framework that brings health governance and climate change together should be considered as a matter, not only of health promotion and disease prevention, but of human and population security. Governance responses should account for the increased cross-border movement of people by addressing health-climate issues within a regional frame that acknowledges and works with transborder social networks and established historical relations.

In order to be successful, this approach must address the needs of the most vulnerable populations, who are disproportionately impacted by the compounded insecurities wrought by extreme weather and climate variability. Because we anticipate that climate impacts will continue to shape mobility patterns of human populations and increase displacement significantly [47], the experiences of populations involved in transborder transhumance will be telling in designing cross-border health and climate governance strategies.

To conclude, our ability to mitigate the synergistic health impacts of climate change relies on the development of creative and comprehensive approaches that transcend many of the epistemic, geographic, economic and political barriers that currently exist. The stakes are high in this endeavor, as climate-related morbidity and mortality rates, as well as population displacement, will undoubtedly continue to grow unless our governance strategies mirror the phenomena they are meant to address.
